# Symphysiotomy

**Published:** 1899-03

**Authors:** Walter C. Swayne

**Affiliations:** Obstetric Physician to the Bristol Royal Infirmary.


### SYMPHYSIOTOMY.

BY

WALTER C. SWAYNE, M.D. Lond.,

*Obstetric Physician to the Bristol Royal Infirmary.*

---

THIS operation was originally proposed by Sigault, a French medical student, in 1768, as a substitute for Cæsarean section, and he performed the first successful operation in 1778, as a result of which it was warmly advocated and often performed for a few years; the first operation in England being performed by Welchman, a Warwickshire surgeon, in 1782. As, however, the results proved disappointing, the maternal mortality being very high and not much inferior to that after Cæsarean section, being about 33 per cent. in the former to 42 per cent.[2] in the latter case, it fell into disuse in a comparatively short time. Being revived by Morisani, of Naples, in 1866, it was practised with greater success, and, the mortality falling greatly on the introduction of antiseptic methods, rose again into favour; and at the present time a large number of cases of recent date are on record.

Originally introduced as a substitute for Cæsarean section, it has now come to be regarded as the proper substitute for craniotomy, since, although the maternal mortality is rather larger, the child, instead of certain destruction awaiting it, has a very good chance of survival. It has also been performed deliberately at term in cases of contracted pelvis instead of the induction of premature labour, and here the results as

[1] Read before the Bristol Medico-Chirurgical Society, December 14th, 1898.

[2] Churchill's *Midwifery*.

regards the life of the fœtus are also very much better, but the maternal mortality is higher. The combined mortality rate in the hands of Pinard and Tarnier[1] respectively is 18.36 for the former in symphysiotomy, and 20.29 for the latter in induced labour. As a substitute for craniotomy it is always performed as an operation of emergency, and it is under this head that the operation described in this paper was performed.

The general indications for its use may be briefly stated as follows:—(1) Absolute contraction of the pelvis when the conjugate diameter is above $2\frac{3}{4}$ inches; (2) relative contraction when the fœtal head, not being enlarged by hydrocephalus, is too large to pass through a normal sized or only slightly contracted pelvis. It is not suitable for cases in which a tumour impacted in the pelvis or bony outgrowth prevents the descent of the head. An essential condition for its performance is, that the child should be alive. The time for operating is when the first stage of labour is complete or almost complete, and the cervix so dilated as to allow of the easy application of the forceps and the passage of the head through it. It should not be performed for rigidity of the soft parts, or when great rigidity is present. Most careful attention to antiseptic measures is necessary, and the patient should as far as possible be prepared as if for an abdominal section, the pubes being shaved and the skin cleansed as perfectly as circumstances will admit. The operation itself is performed by two methods—the subcutaneous and the open methods. The former is the preferable, as the longer the incision in this region the greater the difficulty in preventing contamination of the wound. All that is necessary is, that the incision should be of sufficient length to allow of the free passage of the finger behind the pelvic articulation.

Two assistants are necessary in addition to the anæsthetist, as after section of the joint care must be taken to avoid the too wide separation of the bones during the passage of the head through the pelvis. The dangers to the patient are from hemorrhage and sepsis: the first, however, is easily controlled, and the second should not be likely nowadays. The open method by long incision is said to cause less hemorrhage, since

[1] *Presse méd.*, 1895, 265.

this is usually produced by laceration of the soft parts and vessels around the joint on its separation, but the increased risk of contamination rightly seems to render the subcutaneous method preferable. The most experienced operators do not consider that wiring or suturing the bones together is of any material advantage, and in several cases partial necrosis of the bones and troublesome fistulæ have resulted where this method of treatment has been adopted. The gain in space which is the result of the operation is as follows:—As much as 1¼ inches may be gained as a maximum: that is, when the maximum divergence consistent with the integrity of the sacro-iliac joints is caused, the divergence is just about three inches, and beyond this it should never be allowed to pass. Since the transverse diameter increases very nearly to the same extent as does the distance between the separated ends of the bone, it is obvious what an increase in the sectional area of the pelvic canal can be gained. In the case described the true conjugate was estimated at 2¾ inches; and as the separation of the bones during the extraction was about 2½ inches, nearly an inch was gained in the antero-posterior and about 2¼ inches in the transvere diameters bringing the available space up to that required for an ordinary forceps extraction, namely, to 3¾ inches antero-posterior, and to considerably more than the normal in the transverse, which, although not measured, was obviously contracted, though not to so great an extent as the antero-posterior. The following is the history of the case:—

Mrs. S., aged 25, primipara, at the full term of pregnancy. Labour pains commenced at 3 a.m. on the 22nd of October, 1898. A midwife attended her, and finding that no progress had been made by the evening of that day, administered a grain of opium in pill. Twenty-four hours later, as the head did not advance, she called in a medical man, who, finding on examination that the pelvis was very contracted, advised her removal to the Bristol Royal Infirmary, to which she was admitted on the evening of the 23rd of October. On admission her condition was as follows:—

The patient is a small woman, showing marked signs of rickets. The abdomen, distended by the enlarged uterus, is more than usually prominent, and the fundus uteri reaches to the ensiform cartilage; the pains strong, and recurring every five minutes. Abdominal palpation shows that the fœtus is lying in the third position, the head presenting; the fœtal movements vigorous, and the uterine contractions strong and regular. On auscultation the fœtal heart is heard to the right of the middle line and below the umbilicus, rate about 140 to the minute. The head was felt to be above the brim, and could not be pressed down into the pelvis. On vaginal examination the promontory of the sacrum was easily reached, and the diagonal conjugate was found to be $3\frac{1}{4}$ inches, the os nearly fully dilated, and the soft parts soft and dilatable. The transverse diameter was also found to be lessened, and the ilio-pectineal line within easy reach throughout its whole length. After an attempt to effect delivery with axis-traction forceps—which, as expected, was unsuccessful,—it was decided to attempt delivery by symphysiotomy. After careful cleansing of the external parts and skin, an incision about $1\frac{1}{2}$ inches long was made just above the upper edge of the pubes in the middle line, and carried down for a short distance through the soft parts below the upper level of the symphysis, but not extending more than half an inch below. The incision was carried above through the interval of the tendons of the recti, and a short transverse incision was made to the left side to allow of the introduction of the finger, when the posterior surface of the symphysis was cleared and a sickle-shaped Galbiati's knife introduced, and the separation of the fibro-cartilage commenced above in order to obtain a groove to guide the blade, as the joint was found to be to the left of the middle line. The knife was then carried down the back of the joint and its probe-pointed end made to project against the anterior vaginal wall, the urethra being pressed to one side by means of a sound. The knife was then worked through the joint from below upwards and behind forwards, and the joint divided. The two halves of the pelvis at once sprang apart, separating about an inch, and rather free hemorrhage occurred. This was controlled by iodoform gauze pushed into the wound. The wound was now covered with a sponge-cloth wrung out of hydrarg. perchlor. solution 1—1000. The head immediately descended to a certain extent, and the axis traction forceps were applied. Under steady, but not too strong, traction the head descended, the patient's thighs being supported by two assistants, and delivery effected in about twenty-five minutes. The placenta was expressed twenty minutes later, and a slight laceration of the perineum repaired and the uterus washed out with hot perchloride of mercury solution (1—3000). The superficial wound was sewn up with silkworm-gut sutures, two stitches taking up the fibrous structures on the front of the bone, the packing pressed backwards from between the bones, the bones pressed together, and the sutures tied, the pelvis being brought together by strong traction on the ends of a long piece of unbleached calico bandage passed around the pelvis, the wound dressed with iodoform gauze, and a strong bandage fixed firmly around the pelvis.

The further progress of the case was uneventful. The wound united by first intention, except a small portion at the lower end where the skin adaptation was not quite accurate. The temperature on two occasions reached 100° F. The sutures were removed on the sixth day. The calico support to the pelvis was replaced on the third day by a strong webbing belt tightly buckled around the ilia, while a second belt encircled the trochanters. The catheter was used for the first four days. The patient was allowed out of bed on the twentieth day, and went home on the twenty-eighth day. She has had no disturbance of locomotion, and walked over two miles to be shown at a medical meeting in the seventh week after operation. The child, a male, was born alive, and at birth its condition was as follows: Weight, 8 lbs.; length, 21 ins. Measurements of the head: Maximum vertico-mental diameter, $5\frac{1}{4}$ ins.; sub-occipito bregmatic diameter, $3\frac{3}{4}$; occipito-frontal, $4\frac{1}{2}$; fronto-mental, $3\frac{1}{8}$; biparietal, $3\frac{5}{8}$; bitemporal, $3\frac{1}{4}$; bimastoid, 3. The child, now three months old, weighs 12 lbs., and seems perfectly healthy. The mother was able to nurse it; she did so for six weeks entirely, and still does so partially.

I examined the mother on January 14th, as she complained of some prolapse of the anterior vaginal wall. I found the symphysis with a separation of about $\frac{3}{8}$ inch, apparently perfectly firm, and the conjugate diameter not appreciably altered; otherwise she appeared to be in perfect health.

The conditions under which the operation was performed were extremely favourable. Labour was not so far advanced as to prejudice the mother's chance of recovery; in fact, the os was only fully dilated just before the application of the forceps; plenty of assistants were at hand, a good light and all appliances for carrying out antiseptic and aseptic methods; but at the same time the difficulties which would arise in less favourable circumstances are not insuperable, and the operation itself is not in reality much more difficult than a severe craniotomy, which latter, if a living child is to be operated on, is a most loathsome proceeding. For the very careful notes of the case I am indebted to Mr. A. L. Flemming, resident obstetric officer, and Mr. P. G. Stock.

---

After the reading of Dr. W. C. Swayne's paper, Dr. J. G. Swayne said that about 1777, when the operation was first introduced, his grandfather, Dr. Griffiths, was beginning his medical education, and afterwards practised in this city more than fifty years. Mr. J. C. Swayne, Dr. J. G. Swayne's father, was Dr. Griffiths's apprentice; he passed the College of Surgeons in 1808, and practised for forty years in Bristol, until he retired in 1848. If symphysiotomy had ever been performed here, he would have heard of it from his grandfather, father, or Mr. Richard Smith, the senior surgeon to the Bristol Infirmary. This was almost certainly the first case in which the operation had been performed in this city.

---

